# Eating Like a Rainbow: The Development of a Visual Aid for Nutritional Treatment of CKD Patients. A South African Project

**DOI:** 10.3390/nu9050435

**Published:** 2017-04-28

**Authors:** Cecile Verseput, Giorgina Barbara Piccoli

**Affiliations:** 1 RD Consultant Renal Dietitian, 6 Janet Street, Glenvista, Johannesburg 2091, South Africa; verseput@vanilla.co.za; 2 Department of Clinical and Biological Sciences, University of Torino, 10124 Torino, Italy; 3 Nephrologie, Centre Hospitalier Le Mans, 72037 Le Mans, France

**Keywords:** CKD, diet, nutritional therapy, visual aids, education, empowerment

## Abstract

Providing nutritional education for chronic kidney disease (CKD) patients in South Africa is complicated by several conditions: the population is composed of diverse ethnic groups, each with its own culture and food preferences; eleven languages are spoken and illiteracy is common in the lower socio-economic groups. Food preparation and storage are affected by the lack of electricity and refrigeration, and this contributes to a monotonous diet. In traditional African culture, two meals per day are often shared “from the pot”, making portion control difficult. There is both under- and over-nutrition; late referral of CKD is common. Good quality protein intake is often insufficient and there are several misconceptions about protein sources. There is a low intake of vegetables and fruit, while daily sodium intake is high, averaging 10 g/day, mostly from discretionary sources. On this background, we would like to describe the development of a simplified, visual approach to the “renal diet”, principally addressed to illiterate/non-English speaking CKD patients in Southern Africa, using illustrations to replace writing. This tool “Five steps to improve renal diet compliance”, also called “Eating like a Rainbow”, was developed to try to increase patients’ understanding, and has so far only been informally validated by feedback from users. The interest of this study is based on underlining the feasibility of dietary education even in difficult populations, focusing attention on this fundamental issue of CKD care in particular in countries with limited access to chronic dialysis.

## 1. Introduction

Chronic kidney disease (CKD) is highly prevalent in South Africa, a country in which there is a double burden of under- and over-nutrition [1,2,3,4]. One in four households still experience hunger, while in one in five households the diet is monotonous and low in nutrient diversity, low in proteins with high biological value, and contains high quantity, low quality food, with excessive amounts of cheap starches, fats, and sugars, causing obesity, a known risk factor for CKD [5,6]. Hypertension, often combined with obesity, is the major cause of CKD in South Africa; salt-sensitive primary hypertension often presents at an early age in the native African population [1,2,3,4,7]. Type 2 diabetes, often combined with hypertension and obesity, is the second cause of CKD in South Africa. HIV affects 6% of the population and contributes to HIV-associated nephropathy, another leading cause of CKD [2,8,9].

As in many African countries with high numbers of poor people, late referral of CKD is common. Routine checkups are not part of the African culture and traditional, often nephrotoxic remedies are usually the first choice of treatment, and generally medical assistance is sought only when these cures have failed [8,9].

There are many social factors that make it difficult to convince patients of the importance of the renal diet. High biological value protein intake is insufficient and there are misconceptions about protein sources; these problems are shared by several other African countries [4,10,11]. 

In this predominantly low socio-economic population, most patients’ diets were monotonous, low in high quality protein, and low in vegetables and fruit; due to frequent lactose intolerance, dairy products were generally avoided. Daily sodium intake is usually high and averages 10 g/day, mostly from discretionary sources [4,5,7]. 

In traditional African culture, only two meals per day are consumed, and these are often shared, “eaten from the pot”, thus making it difficult to quantify portion sizes [4,5]. There is often very little variety from day to day, with the exception of Sundays, when a whole chicken is often cooked for the family and served with rice, a green vegetable such as cabbage or spinach, and sometimes a second vegetable; Jell-O, tinned peaches, or packaged custard are usually served as a dessert [4,5]. With Westernized influences, baked bean salad and variations of coleslaw or tuna pasta salad with mayonnaise are increasingly popular and often referred to as a ‘full meal’ in 24 h dietary recalls. 

South Africa is a composite country, with diverse ethnic groups, each having its own culture and food preferences. Eleven languages are spoken, and only about 8% of the population speaks English as a mother tongue. Furthermore, the lower socio-economic groups have a high prevalence of illiteracy, limiting the potential of written educational materials. Lack of electricity and refrigeration contributes to a monotonous diet and low diet diversity [12,13,14].

Educational material is important for improving self-care [15,16,17,18,19,20]. Current studies suggest that discussion between the patient and a member of the health-care team (physician, nurse, or dietitian) remains the preferred method for knowledge transfer regarding CKD; in this regard, educational material can reinforce discussion during clinical encounters. Recent studies recommend a fifth-grade reading level for educational materials for CKD patients; however, while this target may be too low in some settings, it is too high in others, in particular in low-income socio-economic groups, such as the patients to whom our work was addressed [17,18,19]. 

Low literacy is obviously also linked to low health literacy, which has been associated with poorer health related outcomes in CKD patients and families, as well as in patients with other chronic diseases [16,21,22,23,24].

When counseling indigent CKD patients in South Africa on food choices, our aim is to convince them to achieve good-quality protein intake applicable to the patient’s specific CKD stage, and greater food variety, including suitable fruit and vegetables on a daily basis, and a lower intake of discretionary sodium. 

To simplify renal diet education of illiterate or non-English speaking CKD patients in South Africa, we tried to overcome these difficulties by using illustrations, symbols, and colors to replace writing. This was achieved by developing a five-step visual approach to the renal diet, which we called “Eating like a Rainbow”, to try to increase interest and emphasize that following a diet is not necessarily an unpleasant or grim experience. Compliance is ultimately the goal of nutritional intervention in CKD; comprehension is the key for obtaining compliance [21,22,23,24,25,26,27]. The material whose development is here described was developed using a trial-and-error approach over a 4-year period in the renal unit of a large public facility serving lower socio-economic patients without medical insurance. 

The aim of this report is to describe this visual aid as an example of an adaptation of nutritional supports that can be given to CKD patients in a particularly complex socio-economic setting. The interest of this study is based on underlining the feasibility of dietary education even in difficult populations, focusing attention on this fundamental issue of CKD care in particular in countries with limited access to chronic dialysis, both to avoid or retard dialysis needs, and to maximize the advantages of dialysis, for the patients that had access to this treatment.

## 2. Materials and Methods

### 2.1. Study Setting 

The renal unit at the Chris Hani Baragwanath Academic Hospital where the visual tool was developed comprises an “acute section” with four dialysis machines, offering slots for 24 patients that are new admissions, where CKD is suspected but not yet confirmed. Due to the limited access to dialysis in South Africa, acute-injury kidney patients are admitted to the renal unit only if affected by pregnancy-related complications, lupus erythematous, or traumatic injuries. The maintenance hemodialysis unit offers treatment for 81 patients, dialyzed for 4 h three times a week, with a separate section for patients with hepatitis C (treatment for 12 patients). Kidney transplants are not done at this facility. 

Due to the limited access to chronic dialysis available in South Africa, the unit follows the policy of a 6-week evaluation of all incoming patients that need dialysis. Once CKD stage 5 is diagnosed, before considering long-term renal replacement therapy, it has to be established whether the patient is a South African citizen and is eligible for transplantation according to government regulations. Peritoneal dialysis is the first option of renal replacement therapy, provided sufficient eyesight is present (except in cases of previous abdominal surgery, body mass index (BMI) above 32, and/or central obesity); a registered dialysis nurse visits the patient’s home to check that there is enough space for a month’s delivery of dialysate solutions and running water. If patients are not suitable for peritoneal dialysis, hemodialysis is considered depending upon the places available. 

While the availability of dialysis is slowly increasing in South Africa, limited access to treatment makes diet of pivotal importance for assuring survival, and not only for prolonging dialysis-free follow-up. A renal dietitian takes part in the weekly ward visit to new patients, accompanying the nephrologist and the dialysis nurses. 

### 2.2. Development of the Educational Material

The basic idea was very simple, and was developed by the first author of this report, and informally validated by showing it to the nurses and to the patients, within a classical empiric trial-and error procedure.

Our approach was to use illustrations to explain the steps that need to be followed to newly diagnosed, illiterate, non-English speaking CKD patients in a public hospital. We created a question and answer pathway, with five basic questions and illustrated answers: the material used in the illustrations was taken from what is available on the Internet. While this choice is not always elegant, it has the advantage of employing a common visual language, with easily recognizable icons. 

### 2.3. First Step: Choosing the Symbols

**Figure 1:**
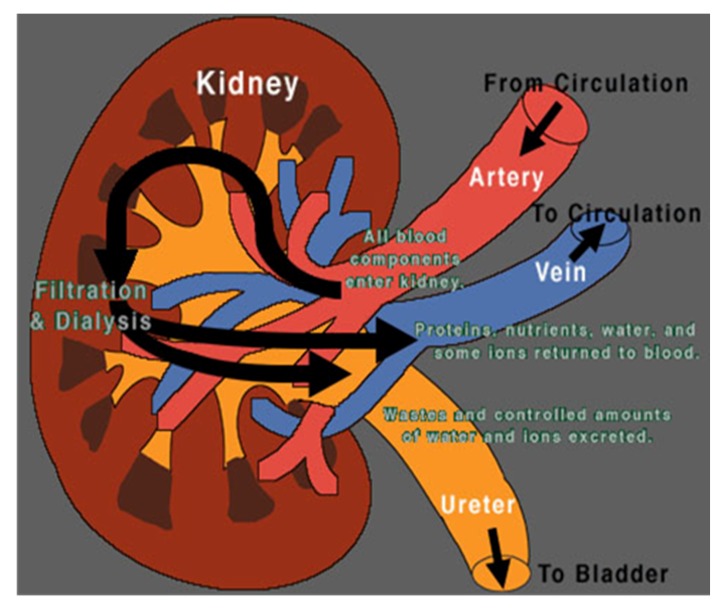
The kidney. Image from [28].

**Figure 2:**
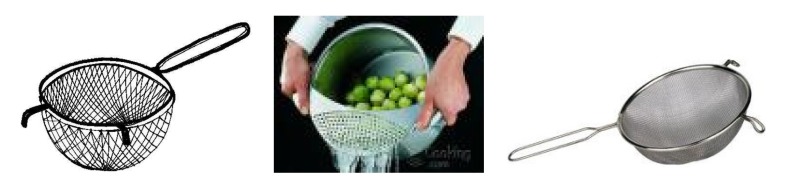
Kidneys work as sieves: these are the images, taken from the Web, that we used to explain kidney function (and malfunction).

**Figure 3:**
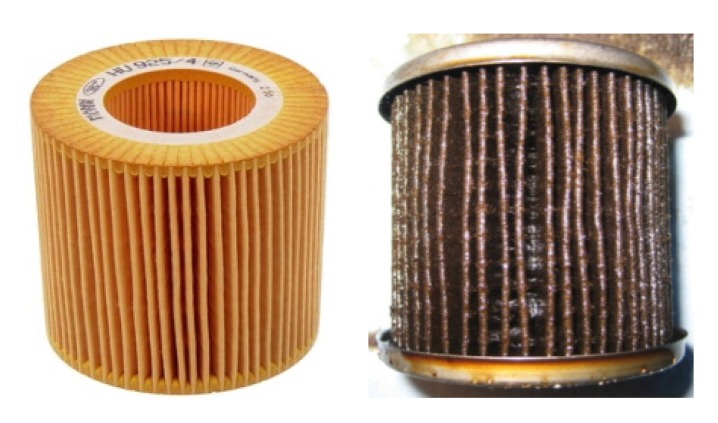
Healthy kidneys clean blood like an oil filter cleans oil in a car. Kidney malfunction is compared to a blocked oil filter, and as a consequence, the body is compared to a car that cannot drive (images taken from the Web).

Due to language barriers and low health literacy, the illustrations used to explain kidney anatomy needed to be very simple. For example, Figure 1, initially chosen, was usually not understood [28]. Therefore, we compared normal kidney function to a sieve with coarse mesh where bigger elements were unable to pass through while water and smaller elements could do so. Impaired kidney function was therefore compared to a blocked sieve. This analogy, shown in Figure 2, was immediately understood by female patients, however, men did not always grasp what was meant, and so, considering the shape of the filter on the dialysis machine, we compared kidney function to a clogged oil filter, which causes damage to a car (Figure 3). 

**Figure 4:**
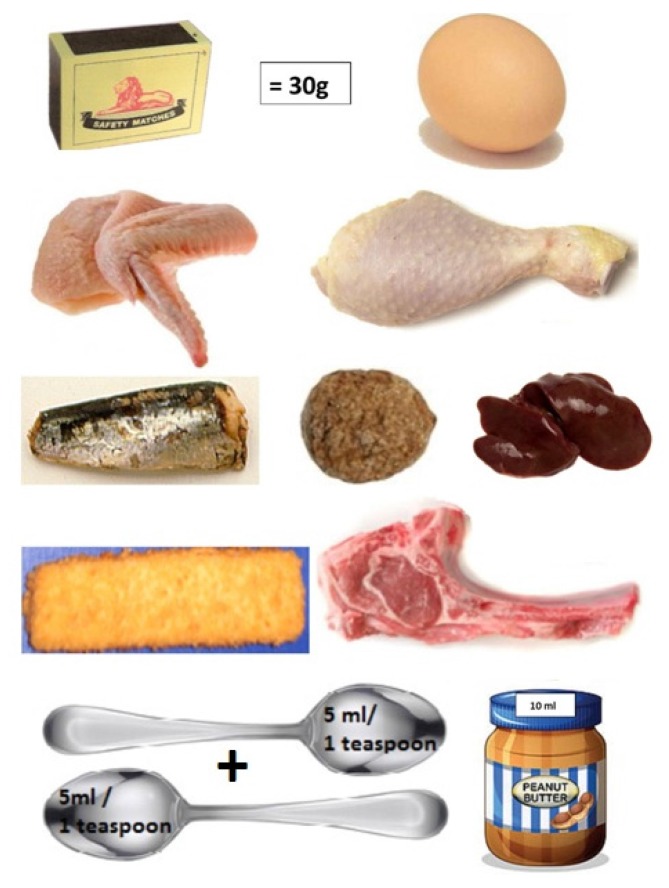
Protein portions are compared to a match box: one match box = 30 g and contains 7 g protein.

To help patients understand suitable portion sizes, meat portions are compared to a matchbox (Figure 4), fruit to a tennis ball, and starches to a fist.

**Figure 5:**
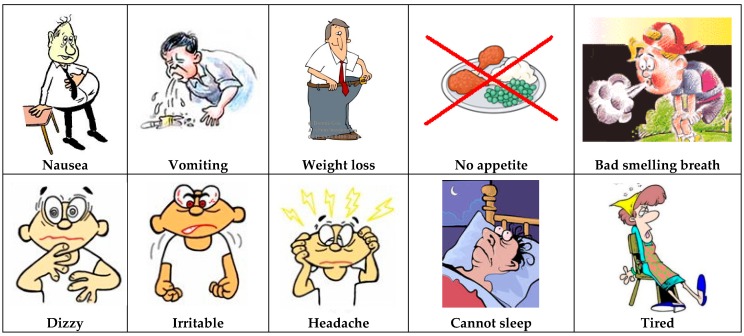
Urea intoxication: the concept of “uremia”.

The explanation of the role of urea and retained toxins followed the concept of the clogged sieve and of sodium and water: urea was depicted as a poison causing uremic symptoms like nausea and vomiting (Figure 5). The following point was that other chemicals accumulated and caused symptoms: the two most important ones were potassium and phosphate. The role of the dialysis machine became clear: the kidney is damaged (sieve is clogged) and the machine cleans the blood. It was also always mentioned that the machine was giving the kidneys time to rest in order to recuperate if still possible. 

### 2.4. Second Step: Choosing the Sequence

The five-steps approach used in the educational pathway was defined by discussion within the caregivers’ team through analyzing the most common needs and situations. In our experience, the best starting point was referring to the symptoms experienced on admission. Since most patients are edematous when they arrive at the hospital, we started by explaining how this symptom is caused by kidney damage. 

The sequence that was found to work better in the clinical practice was as follows:

Step 1. What foods do you eat every day? This is the basis for obtaining the patient’s diet history, and for introducing the illustrated material. 

Step 2. How do the kidneys work? Normal kidney function is compared to a sieve or oil filter and the damaged kidney to a blocked sieve or oil filter, causing waste and electrolytes to be retained and leading to edema and intoxication, symptoms frequently experienced by patients on admission to the hospital. 

Step 3. What should you eat? A “renal plate” was designed, trying to focus on the most popular food in Southern Africa, showing the three food groups and suitable food choices for each group. If the monthly blood results reflect elevated electrolytes, or the patient was overloaded when referred to the renal clinic, patients would be referred to Step 4. 

Step 4. What foods cannot be eaten? The symptoms experienced in CKD, for example edema, are linked to a culprit, such as sodium. Food rich in potassium, phosphates, and sodium are illustrated, and each one is marked with an X. Unsuitable fluids are also illustrated. 

Step 5. How much should be eaten? This step is not always implemented with precise portion sizes (grams or milliliters); in fact, measuring and weighing foods is a concept foreign to African culture. 

### 2.5. Third Step: the Renal Plate

Suggestions from the literature were obtained whenever possible; while the “five step” approach was gathered into a simplified classic “booklet like” approach, a renal plate, adapted to South African patients, was built by adapting similar approaches found in the literature and on the Internet [29,30,31]. Due to the problems in defining portions to our patients, our choice was to adapt the plate to a qualitative reading, and to include allowed and forbidden food using the same visual tool.

## 3. Results

### 3.1. The Five-Step Approach

The complete pathway for the five steps (What foods do you eat every day? How do the kidneys work? What should you eat? What foods cannot be eaten? How much should be eaten?) is usually completed in more than one visit: most patients are in a full-blown uremic syndrome on admission and two to three visits are needed to discuss all these issues. 

**Figure 6:**
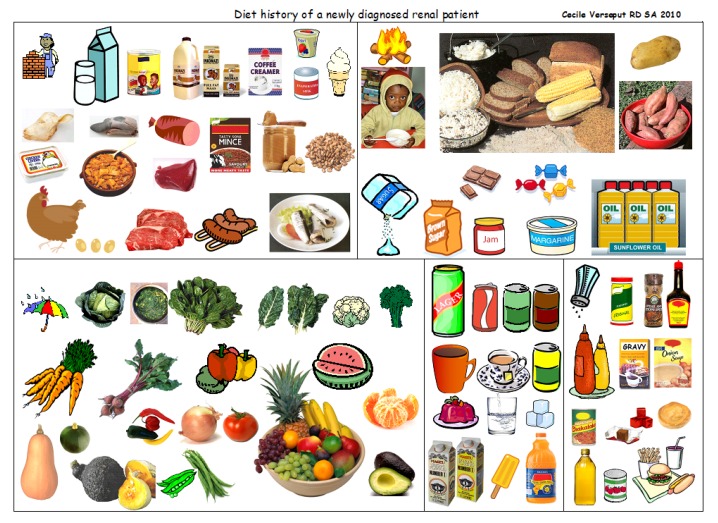
A visual tool, supporting the recall of the diet history (images taken from the Web).

Figure 6 was used to help obtain a diet history; the dietitian would indicate a picture and the patient would choose by a thumb up for yes, and down for no. This page is a relatively comprehensive collection of the foods most frequently eaten: dietary variety is indeed limited, and this eases the task of taking a diet history. 

Patients usually think in terms of cooked food, while dietitians think in terms of nutrients. The renal dietitian needs to link nutrients to foods to help patients understand that certain nutrients from certain foods can affect body functions in CKD.

**Figure 7:**
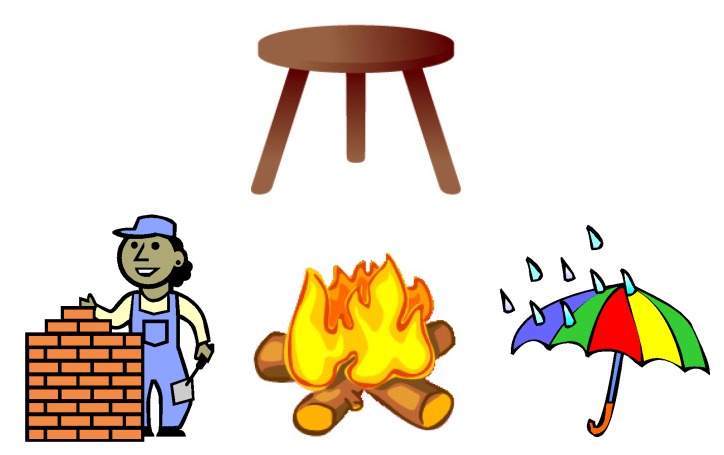
The three-legged chair compared to the body building foods (bricks), the energy foods (fire), and the protective foods (umbrella).

We tried to achieve this goal by dividing food into three main groups: body building foods (compared to bricks); energy foods (compared to fire); and the protective foods (compared to an umbrella). These were further compared to a three-legged chair: the three food groups are needed to maintain health, just like the chair needs its three legs to be functional (Figure 7).

**Figure 8:**
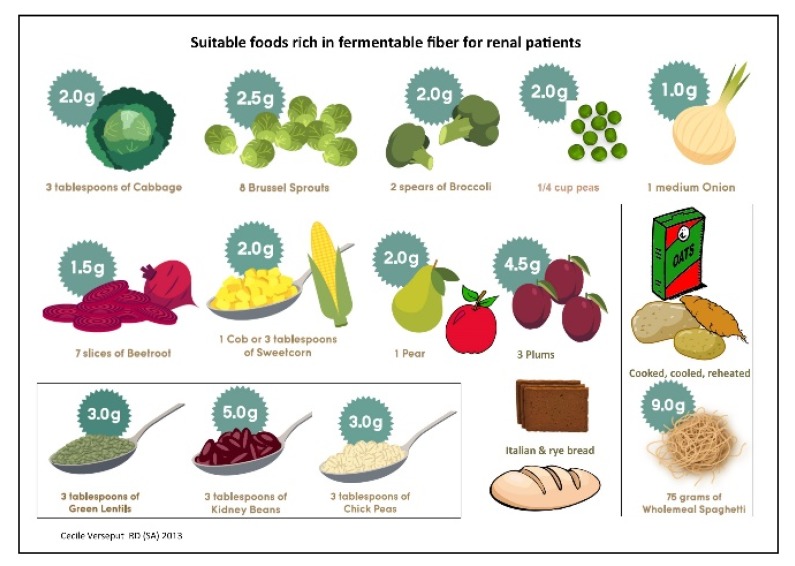
Food with renal friendly fiber.

These concepts were gathered in a four-page booklet. The first page depicts the body building and protective foods that have to be chosen first: affordable sources of good quality protein, as the first obstacle was to include sufficient, good quality protein in each CKD stage; a second step was emphasizing that consuming too little or too much are both risks. Fruit and vegetables have an important protective function and the need to include these renal friendly options on a regular basis was underlined (Figure 8).

Patients generally inquired about their favorite fruits and vegetables: the second page was dedicated to this topic. The figures also explain that fruits and vegetables that are rich in potassium could cause symptoms such as irregular heartbeat, fainting, and cardiac arrest if too much is eaten, and should be avoided. 

Page three deals with energy foods to include, drinks that are allowed and ones to avoid. It also explains that 500 mL of fluid plus output makes up the usual daily fluid allowance.

Page four deals with sodium: the patient is reminded about the symptoms they manifested on admission, partly caused by high sodium intake. The biggest problems are caused by adding salt during cooking and at the table, and by the extensive use of high sodium condiments (some of which do not taste salty), processed meats, ready-to-eat foods, and snacks. The double burden of phosphate is also discussed.

### 3.2. The Renal Plate

**Figure 9:**
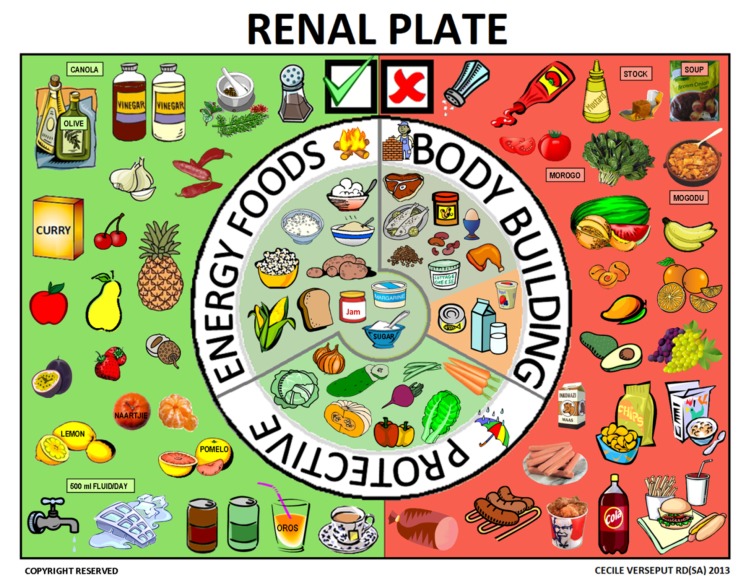
The renal plate.

The “renal plate” was the solution chosen to explain food groups and allowed and forbidden foods on the same page (Figure 9). The explanation started with the “body building foods”, followed by suitable “energy foods” and “protective foods”. 

This format is not new: the plate has been used in several settings, from the well-known “ChooseMyPlate” in the United States, divided into four parts, to the three-part Canadian plate (where half is vegetables and fruits), to various versions of vegan or vegetarian plates [29,30,31]. 

Plates and pyramids, which are probably even more widely used, convey a similar message: how much of each food group should be eaten. Pyramids are probably more easily understood in Europe and the U.S. and convey a hierarchy of food that should be eaten when healthy or suffering from an illness [32,33,34]. In South Africa, however, we thought that the pyramid might be interpreted differently, for instance as “the best is at the top”. Unlike the plates and pyramids cited above, the renal plate described here is used to support a qualitative rather than a quantitative message, based on the three food groups that need to be consumed daily and showing also forbidden foods (red background for forbidden ones, green background for allowed ones).

This format proved to be easily understood: it was visually striking, clearly organized, and simple. When confronted with the renal plate, the patient was usually still hospitalized and received food from the hospital kitchen. The red section was therefore only applicable after hospital discharge, but could also help in choosing food from what was brought to the hospital by the patient’s family. 

Once the patient was familiar with the renal plate, he or she usually started asking about foods not shown on the plate; therefore, the next steps included discussion on different foods, taking the patient’s background into account, and additional sections on fluids, spices, and herbs, were presented on separate pages. This detailed information was usually given when the patient had been considered for chronic renal replacement therapy, and usually took place during peritoneal dialysis (PD) training, thus after six weeks. One of the aims of the manual is to change the patient’s mind from thinking there is no food left to eat to giving guidance for better choices, and to help patients to avoid thinking that the diet is worse than the disease. 

**Figure 10:**
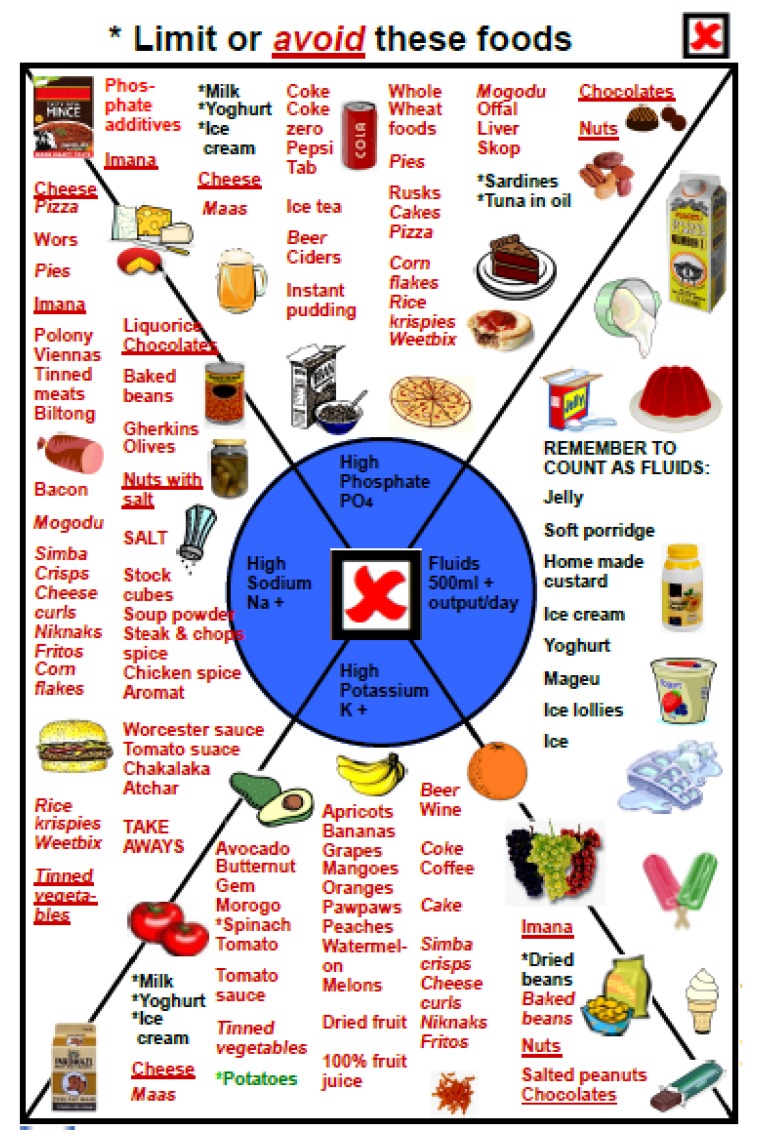
Food to limit or avoid.

A specific section was developed with detailed information on foods that could not be eaten (a red X on each page), and was mostly used in the PD outpatient clinic, when the nephrologist referred the patient for a specific problem, like high potassium or phosphate levels or uncontrolled blood pressure. In this section, the patient was reminded about the link between symptoms and food; for example. irregular heartbeat was linked to potassium levels, with illustrations of high-potassium foods, and a written diet sheet was usually given to the patient to take home (Figure 10).

Because most patients prior to admission only ate two meals per day, they were counseled to divide their food into smaller portions, especially if their appetite was poor. Once the patient was estimated to have achieved a good diet balance, a second diet history was taken in the PD clinic, once more addressing the possible errors, and trying to address portion sizes. Two visual files were developed, with a 60 g- and 80 g-protein diet.

All these separate files were finally condensed into a five-step booklet, entitled “Five illustrated steps to improve renal diet compliance”, for use as a tool when educating CKD patients. 

**Figure 11:**
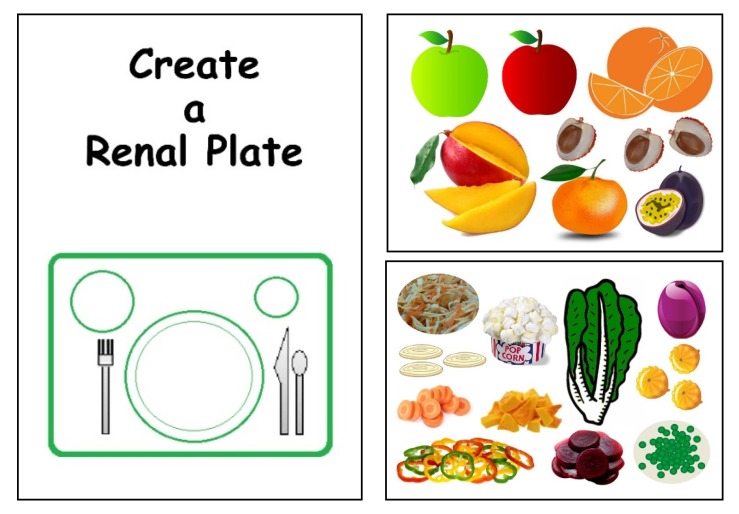
Create your renal plate: an educational game.

Furthermore, the educational game: “Create your renal plate” (Figure 11) was initially developed for children, but proved to be useful also with adult patients: it consists of 10 pages with color illustrations of food in all the food groups. These illustrations need to be cut out to build a plate, and the game provides useful information on eating habits, reflected in different food choices. 

## 4. Discussion

In CKD, besides the usual approaches to reducing complications and progression of the disease, such as blood pressure and glycemic control, changes in diet and lifestyle are vital. As Tuot pointed out, “Implementing CKD risk-modification behaviors requires not only clinician awareness, but also patient understanding and engagement in health. Offering educational materials to patients is an important strategy” [18]. A recent review that examined almost 100 different educational tools found that few were actually easily readable by the patients [19]. This may be particularly true in some settings, such as in South Africa, where many adults lack the skills required to read, understand, and engage in effective self-management or have a “below basic” level of “prose literacy”, defined as the ability to use “printed and written information to function in society, to achieve one’s goals, and to develop one’s knowledge and potential” [26].

Developing appropriate patient education materials poses challenges everywhere in the world, and is even more difficult in countries such as South Africa, where numerous cultures and languages are present and a large part of the population is of low socio-economic status. Having a visual tool with limited wording also meant not having to translate into the numerous languages spoken in South Africa.

The problem of illiteracy is, however, more widespread that often considered, and industrialized countries are not spared by this “silent epidemic”, as a report on the New England Journal of Medicine recalls in the new millennium [35].

Simple visual aids may be of great help not only in recalling diet-related concepts, but also in reassuring patients, thus ensuring empowerment and compliance even in difficult settings [4,36].

Renal diet education needs time, skills, and requires organization and is extremely context sensitive: the same diets may not be successful in Australia, Italy, Brazil, or India, but reports of successful implementation may come from all continents [37,38,39,40]. 

Due to the scarce availability of dietetic services in remote areas, training is often extended to all the professional caregivers of CKD patients, and simple, comprehensive materials, such as the ones we developed in our hospital, are needed. The experience reported in this paper suggests that education on the diet is indeed also possible in difficult settings and that simple approaches, such as making “collages” of images found on the Internet, may represent useful, simple, and inexpensive tools to enhance comprehension.

The educational material presently does not cover several fundamental issues that are routinely discussed with the dietitians, but not yet integrated in the developed material: smoking discontinuation is a must in CKD patients, and it is an important problem in several developing countries, including South Africa [41,42,43]. Correction of acidosis, as well as maintenance of regular physical activity and correction of obesity are two further fundamental issues; a plant-based approach may be of help, in particular for acidosis correction [44,45,46,47,48]. All these issues merge into the need for higher food-awareness, a comprehensive concept that needs to be adapted to the different clinical and cultural contexts [49]. The educational material is indeed a work-in progress, and it is available upon request to the authors.

The books, booklets, and leaflets described in this report have not been formally validated. This is a major limitation of our report, which deals with opinions based on direct experience, whose effect in improving knowledge and changing lifestyle still needs to be quantified. Validation is our next goal, and we hope that the present report will help us to obtain the funds needed for a dedicated study, since our aim is also to raise awareness and increase investment in these fundamental aspects of renal care. 

In this regard, due to the difficulties in differentiating the effect of the education from that of the diet by itself and of the “global care” of patients who often were never previously treated for CKD, we will in the first step involve the renal dietitians operating in South Africa in a questionnaire based survey, and, according also to their opinions, a patient centered prospective survey will follow.

However, within these limitations, we considered that the approach we developed could be of interest in the world-wide panorama of ways being used to provide nutritional care of CKD, the difficulties that may be encountered, and the costs that are involved. 

## 5. Conclusions

In conclusion, the experience described in this report underlines the difficulties, but also the feasibility of an educational approach to low income, low literacy populations, and supports a creative use of common tools, such as Internet derived images, to develop educational material targeted to the cultural, educational, and clinical characteristics of the population studied.
